# Progression prediction of coronary artery lesions by echocardiography-based ultrasomics analysis in Kawasaki disease

**DOI:** 10.1186/s13052-024-01739-1

**Published:** 2024-09-18

**Authors:** Dan Xu, Chen-Hui Feng, Ai-Mei Cao, Shuai Yang, Zhen-Chao Tang, Xiao-Hui Li

**Affiliations:** 1 Department of Cardiology, Children’s Hospital Capital Institute of Pediatrics, No.2, Yabao Rd, Chaoyang District, Beijing, 100020 China; 2 Graduate School of Peking Union Medical College, Chinese Academy of Medical Sciences, Beijing, China; 3 Capital Institute of Pediatrics-Peking University Teaching Hospital, Beijing, China; 4 Beijing Advanced Innovation Center for Big Data-Based Precision Medicine, School of Engineering Medicine, Beihang University, 37 Xueyuan Road, Haidian District, 100191 Beijing, China; 5 Key Laboratory of Big Data-Based Precision Medicine, Beihang University, Ministry of Industry and Information Technology of the People’s Republic of China, Beijing, China

**Keywords:** Kawasaki disease, Coronary artery lesions, Echocardiogram, Ultrasomics, Pediatric

## Abstract

**Background:**

Echocardiography-based ultrasomics analysis aids Kawasaki disease (KD) diagnosis but its role in predicting coronary artery lesions (CALs) progression remains unknown. We aimed to develop and validate a predictive model combining echocardiogram-based ultrasomics with clinical parameters for CALs progression in KD.

**Methods:**

Total 371 KD patients with CALs at baseline were enrolled from a retrospective cohort (cohort 1, *n* = 316) and a prospective cohort (cohort 2, *n* = 55). CALs progression was defined by increased Z scores in any coronary artery branch at the 1-month follow-up. Patients in cohort 1 were split randomly into training and validation set 1 at the ratio of 6:4, while cohort 2 comprised validation set 2. Clinical parameters and ultrasomics features at baseline were analyzed and selected for models construction. Model performance was evaluated by area under the receiver operating characteristic curve (AUROC), area under the precision-recall curve (AUPRC) and decision curve analysis (DCA) in the training and two validation sets.

**Results:**

At the 1-month follow-ups, 65 patients presented with CALs progression. Three clinical parameters and six ultrasomics features were selected to construct the model. The clinical-ultrasomics model exhibited a good predictive capability in the training, validation set 1 and set 2, achieving AUROCs of 0.83 (95% CI, 0.75–0.90), 0.84 (95% CI, 0.74–0.94), and 0.73 (95% CI, 0.40–0.86), respectively. Moreover, the AUPRC values and DCA of three model demonstrated that the clinical-ultrasomics model consistently outperformed both the clinical model and the ultrasomics model across all three sets, including the training set and the two validation sets.

**Conclusions:**

Our study demonstrated the effective predictive capacity of a prediction model combining echocardiogram-based ultrasomics features and clinical parameters in predicting CALs progression in KD.

**Supplementary Information:**

The online version contains supplementary material available at 10.1186/s13052-024-01739-1.

## Key message

Early identification of patients at risk of progression of coronary artery lesions remains vital for improving the prognosis of patients with Kawasaki disease. Whether ultrasomics help predict the progression of coronary artery lesions in Kawasaki disease remains unclear. The present study included 371 patients with Kawasaki disease and analyzed 1484 echocardiographic images. An integrated model combining echocardiogram-based ultrasomics features and clinical parameters was established and validated, demonstrating satisfactory performance in predicting the progression of coronary artery lesions in Kawasaki disease.

**Supplementary Information:**

The online version contains supplementary material available at 10.1186/s13052-024-01739-1.

## Introduction

Kawasaki disease (KD) is an acute vasculitis of childhood that mainly affects children under 5 years old, and it is the primary cause of acquired heart disease in children of developed countries [1]. Globally, the incidence of KD has substantially increased over the past decades [2], which was approximately 107.3 per 100,000 children aged < 5 years in China [3]. Coronary artery lesions (CALs) are the predominant adverse complications of KD, which consist of coronary artery dilation (CAD) and coronary artery aneurysms (CAA). Despite the widespread adoption of standard treatment, approximately 9.0-15.9% of patients with KD may develop CALs [3–5]. As research on KD progresses, the evolving research focus has shifted from merely assessing CALs’ occurrence to monitoring their changes in the course of disease [6–8].

Several studies reported that 77.4–82.0% CALs normalize in dimensions within 2 years after KD onset [9, 10], roughly 24% of patients with initially diagnosed CALs may persist or progress in subsequent evaluations [11]. Notably, progressive CALs correlate with adverse late coronary artery outcomes [12], warranting heightened attention for KD patients experiencing CALs progression. Since primary adjunctive treatment can ameliorate the coronary artery outcomes of KD patients with CALs [13, 14], early identification of patients at risk of CALs progression remains vital for improving their prognosis.

Utilizing intricate computer algorithms to extract extensive data from images, ultrasomics can analyze numerous quantitative image features that are challenging to discern with the naked eye [15, 16]. Increasing studies have indicated the potential of radiomics in diagnosing and predicting cardiovascular diseases in multiple imaging methods, such as coronary computed tomography angiography (CCTA) [17, 18],cardiac magnetic resonance (CMR) [19, 20], and echocardiographic examinations [21, 22]. As stated by the American Heart Association (AHA), transthoracic echocardiography (TTE) has been recommended as the primary method for coronary artery assessment in KD patients [1]. Recently, two researches have applied deep learning (DL) algorithms to detect CALs on echocardiographic images, aiding in KD diagnosis [23, 24]. However, studies employing echocardiographic images via ultrasomics to identify KD patients at risk of CALs progression have not been reported. Numerous previous studies have explored risk factors for the persistence or progression of CALs based on medical records [6, 9, 25, 26], identifying their associations with coronary artery imaging findings such as CAA size at diagnosis and the number of involved coronary arteries [9, 26]. However, there remains a relatively unexplored domain in comprehensively investigating coronary artery imaging features through ultrasomics.

Therefore, we conducted this study to develop and validate a predictive model that combined clinical parameters and echocardiographic ultrasomics features, expecting to identify KD patients at-risk in time and improve the coronary artery outcomes of these children.

## Methods

### Study patients and design

This observational study was conducted at the Department of Cardiology, Children’s Hospital Capital Institute of Pediatrics in Beijing, China. Ninety-six patients were excluded due to various reasons such as incomplete medical records and poor quality of echocardiograpghic images, leaving 371 patients for subsequent analysis. The present study consisted of a retrospective cohort (cohort 1, *n* = 316) and a prospective cohort (cohort 2, *n* = 55). Cohort 1 included KD patients who hospitalized in our center between April 2018 and May 2022 to train and validate of predictive models. Cohort 2 prospectively recruited eligible patients from June 2022 to June 2023 to assess the generalizability of the model.

The inclusion criteria were as follows: (i) confirmed diagnosis of KD; (ii) hospitalized children; (iii) detected with CALs on any of the coronary arteries at the time of KD diagnosis or before intravenous immunoglobulin (IVIG) treatment. The exclusion criteria were as follows: (i) recurrent KD; (ii) no IVIG treatment during hospitalization; (iii) no available follow-up echocardiographic evaluation at 1-month after KD onset; (iv) coexisting congenital heart diseases; (v) subsequent diagnosis of other diseases, such as Takayasu arteritis. (vi) the delineation of regions-of-interest (ROIs) was restricted by the poor quality of the images.

The study was reviewed and approved by the Institutional Research Board of Children’s Hospital Capital Institute (SHERLL2023048). Informed consent was obtained from at least one parent or guardian for each patient.

### Data collection, echocardicgraphic coronary artery evaluation

Each patient’ demographic information, clinical characteristics, responsiveness to IVIG treatment, laboratory indicators prior to the treatment of IVIG, and baseline echocardiographic images (the echocardiogram obtained at the time of diagnosis or before IVIG treatment) were collected. Besides the diagnosis and treatment of KD, the definition of IVIG resistance, definition of complete and incomplete KD, and the frequency of echocardiographic evaluation were all based on the criteria of AHA (2017) [1].

The coronary artery findings were obtained by experienced ultra-sonographers with the Philips ie33 or 7c system. The internal dimensions of left main coronary artery (LMCA), left anterior descending artery (LAD), left circumflex artery (LCX), and 3 segments (proximal, middle, and distal) of right coronary artery (RCA) were measured and recorded by echocardiography and converted to Z scores according to the criteria of Kobayashi Z-score adjusted for body surface area [27]. The maximum Z score (Zmax) was defined as the largest Z score of the four coronary artery branches (LMCA, LAD, LCX, or RCA) on echocardiography.

Given the significant association between the severity of CALs one month after disease onset and late coronary artery outcomes in KD patients [28, 29], the present study compared the Z scores of coronary arteries at the 1-month follow-up with their baseline scores. Patients were categorized into 2 groups based on changes in Z scores of coronary arteries between baseline and 1-month follow-up: (1) CALs-progressed: any of four coronary artery branches presented an increased Z score at the 1-month follow-up; (2) CALs-improved: no coronary arteries presented increased Z scores, and at least one coronary showed a reduced Z score at the 1-month follow-up.

### Image screening, ROIs segmentation and feature extraction

Considering the lowest frequency of occurrence and poor clarity of images regarded the sites of CALs located in the left circumflex and distal RCA, the images of LMCA, LAD, the proximal segment of RCA (RCAp), the middle segment of RCA (RCAm) were selected to subsequent analysis. All echocardiographic images in the DICOM format were anonymized to protect the privacy of the included patients.

Then the ROIs were manually segmented by an ultrasonographer (Shuai, Yang) and confirmed by another experienced ultrasonographer who had over 15 years’ experience (Ai-Mei, CAO) in pediatric cardiology, using ITK-SNAP (version 3.8, www.itksnap.org) software. The ROIs included the vascular walls and lumen diameter of the typical sites of CALs on the images. Both ultrasonologists were blinded to the diagnosis results during the process of cardiac evaluation and ROIs segmentation. Ultrasomics features were extracted in Python (version 3.8.8) using Pyradiomics (version 2.2.0), which complies with the Imaging Biomarker Standardization Initiative (IBSI) guidelines. Intraclass correlation coefficient (ICC) was calculated to assess the reproducibility of feature extraction,  of which an ICC value lower than 0.75 was removed.

### Selection of ultrasomics features

Feature selection was conducted based on the training cohort. All extracted ultrasomics features from each patient were normalized using the Z-score method. Hierarchical analysis was performed based on Pearson’s correlation analysis, and the redundancy features with correlation coefficients > 0.90 were eliminated. Subsequently, an analysis of variance (ANOVA) F-test statistic was used to select the top 30% features ranked by F-value (each feature has a individual F-value related to target events).

Nine machine learning algorithms were trained, including random forest (RF), support vector machine (SVM), decision tree, K-nearest neighbors (KNN), gradient boosting machine (GBM), light gradient boosting machine (LightGBM), extreme gradient boosting machine (XGBoost), multi-Layer perceptron (mLP), bernoulli naive Bayes (bNB). The performance of each algorithm was evaluated from two aspects, including accuracy (ACC) and the area under the receiver operating characteristic curve (AUROC). The optimal algorithm was selected after comprehensive evaluation of above two aspects.

To narrow the range of contributing factors, the importance of each factor was calculated using the SHAP (SHapley Additive exPlanation) tool. After ordering the importance of all variables from the highest to the lowest, the prediction performance of an increasing number of top factors was appraised by ACC, precision, and AUROC, upon which the minimal number of important variables was determined.

### Selection of clinical parameters

The demographic and clinical characteristics, the responsiveness to IVIG treatment, and laboratory indicators prior to the treatment of IVIG of enrolled patients in the training set were analyzed to select clinical parameters for CALs progression. The bidirectional stepwise approach was adopted, and those significant variables (*P* < 0.1) were selected to subsequent model construction.

### Statistical analysis

The clinical model was constructed by the clinical parameters selected by bidirectional stepwise approach using logistic regression analysis. The ultrasomics model was established with the ultrasomic features selected in the way as mentioned above. Finally, a clinical-ultrasomics predictive model was developed by integrating the selected clinical parameters and the ultrasomic features using logistic regression. Moreover, the performance of the three models was assessed by AUROC, area under the precision-recall curve (AUPRC) and decision curve analysis (DCA) in the training and two validation sets.

Results were expressed as numbers and percentages for categorical variables, and median (with inter-quartile range) or mean (with 95% confidence interval) for quantitative ones. Comparisons were performed using the Fisher’s exact test for categorical variables and the Mann-Whitney U test for quantitative variables. A two-sided p-value < 0.05 was considered statistically significant. All data were collected anonymously.

The statistical handling was done by the community PyCharm (edition 2.2.0) on the Windows 10 system with the Python software (version 3.8.8). Missing data were supplemented according to the multiple imputation procedure, which was implemented by the MICE package in the R programming environment (Version 4.0).

## Results

### Demographic and clinical characteristics of enrolled patients

**Fig. 1:**
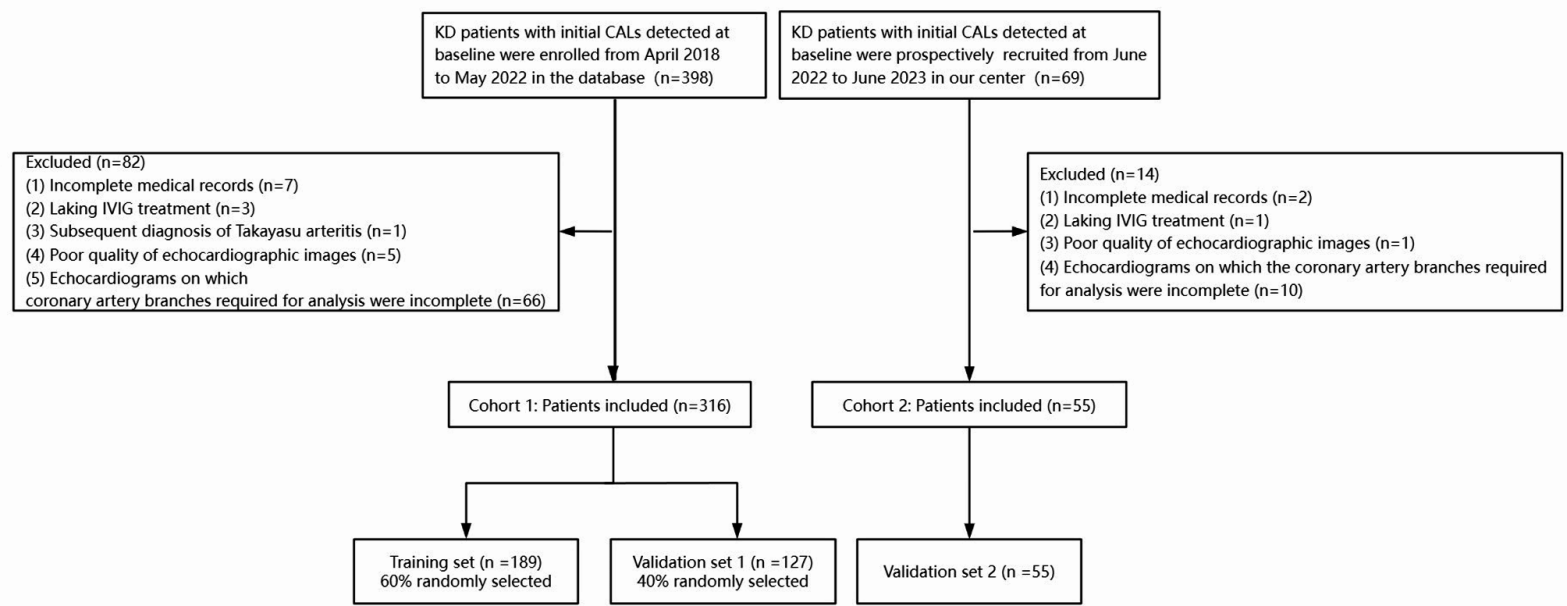
The flowchart of the study. *Abbreviations* KD, Kawasaki disease; CALs, coronary artery lesions; IVIG, intravenous immunoglobulin

As the flowchart of patient selection process shown in Fig. 1, a total of 371 patients were eventually enrolled based on the inclusion and exclusion criteria. Patients in the retrospective cohort were randomly divided into a training set (*n* = 189) and a validation set (validation set 1, *n* = 127) at a ratio of 6:4, whereas patients in the prospective cohort were all included into the prospective validation set (validation set 2, *n* = 55). The baseline characteristics of all participating students are presented in Table 1. At the 1-month follow-ups, 65 (17.5%) patients were categorized into CALs-progressed group, while 306 (82.5%) patients were categorized into CALs-improved group. Except for the significantly higher concentrations of serum fibrinogen (FIB) and interleukin-6 (IL-6) in validation set 2 compared to both the training set and validation set 1 (both *P* < 0.05), no other significant differences were observed among the three sets, indicating that the basically balanced data distribution among three datasets.

**Table 1:** Baseline clinical characteristics of the training and validation sets

Characteristics	Total (*n* = 371)	Training set (*n* = 189)	Validation set 1 (*n* = 127)	Validation set 2 (*n* = 55)	*P*
Demographic Information					
**Age**,** (months) (%)**	19.00 [11.00, 34.00]	20.00 [11.30, 35.00]	17.00 [9.29, 33.00]	19.00 [11.77, 35.00]	0.443
**Sex (%)**					
male	230 (62.0)	120 (63.5)	76 (59.8)	34 (61.8)	0.806
female	141 (38.0)	69 (36.5)	51 (40.2)	21 (38.2)	
**Clinical Information**					
**Fever duration (days)**	6.00 [5.00, 7.00]	5.00 [5.00, 7.00]	6.00 [5.00, 7.00]	6.00 [5.00, 7.00]	0.376
**Initial IVIG resistance(%)**					
**+**	37 (10.0)	21 (11.1)	11 (8.7)	5 (9.1)	0.754
**-**	334 (90.0)	168 (88.9)	116 ( 91.3)	50 (90.9)	
**KD subtypes**,** No. (%)**					
cKD	332 (89.5)	157 (90.8)	127 (88.8)	48 (87.3)	0.802
iKD	39 (10.5)	16 (9.2)	16 (11.2)	7 (12.7)	
**CALs status**					
**CALs progressed**					
Yes	65 (17.5)	40 (21.2)	18 (14.2)	7 (12.7)	0.166
No	306 (82.5)	149 (78.8)	109 (85.8)	48 ( 87.3)	
**Baseline Zmax**	2.63 [2.31, 3.48]	2.70 [2.30, 3.53]	2.58 [2.32, 3.34]	2.59 [2.28, 3.50]	0.845
**NCAI**					
1	189 (50.9)	91 (48.1)	73 (57.5)	25 (45.5)	0.389
2	96 (25.9)	52 (27.5)	28 (22.0)	16 (29.1)	
3	57 (15.4)	32 (16.9)	14 (11.0)	11 (20.0)	
4	29 (7.8)	14 (7.4)	12 (9.4)	3 (5.5)	
**Laboratory Examination**					
WBC (*10^9^/L)	15.90 [13.06, 19.68]	16.32 [13.79, 19.70]	15.41 [12.73, 19.13]	14.92 [11.71, 19.88]	0.115
N%	0.69 [0.60, 0.79]	0.69 [0.60, 0.79]	0.68 [0.61, 0.79]	0.68 [0.59, 0.75]	0.544
Hb (g/L)	115.71 (12.82)	115.93 (13.13)	115.43 (12.85)	115.64 (11.85)	0.942
PLT (*10^9^/L)	319.00 [258.00, 405.50]	306.00 [256.00, 391.00]	336.00 [276.00, 422.00]	322.00 [252.00, 403.50]	0.214
CRP (mg/L)	72.00 [43.00, 104.50]	71.00 [41.00, 106.00]	67.00 [44.00, 100.00]	74.52 [46.79, 107.35]	0.478
ESR (mm/60min)	62.00 [46.00, 80.00]	62.00 [47.00, 81.00]	61.00 [43.00, 80.00]	61.00 [43.50, 81.50]	0.517
Na (mmol/L)	136.00 [133.00, 138.00]	135.00 [133.00, 138.00]	136.00 [133.50, 138.00]	135.00 [133.00, 137.00]	0.306
ALB (g/L)	34.70 [32.20, 37.10]	34.30 [31.80, 36.70]	34.70 [32.85, 37.25]	35.10 [32.50, 37.50]	0.11
Tbil (umol/L)	5.10 [3.70, 8.40]	5.10 [3.40, 8.70]	5.30 [3.95, 8.35]	5.40 [3.95, 7.15]	0.652
NT-proBNP (pg/ml)	602.70 [252.25, 1,444.00]	613.80 [253.10, 1,337.00]	553.60 [223.60, 1,202.50]	806.40 [315.80, 2,021.50]	0.281
PT (s)	12.20 [11.50, 12.90]	12.20 [11.50, 12.90]	12.10 [11.40, 12.85]	12.50 [11.60, 12.95]	0.302
**FIB (g/L)**	**4.92 [4.38**,** 5.84]**	**4.96 [4.52**,** 5.85]**	**4.79 [4.30**,** 5.62]**	**5.34 [4.57**,** 6.04]**	**0.037**
APTT (s)	30.30 [27.90, 33.00]	30.30 [27.90, 33.30]	30.00 [27.55, 32.50]	30.60 [28.55, 33.15]	0.359
D-Dimer (ug/L)	1.15 [0.65, 2.08]	1.16 [0.72, 2.07]	1.00 [0.56, 2.06]	1.20 [0.68, 2.25]	0.304
INR	1.06 [1.00, 1.13]	1.06 [1.00, 1.13]	1.05 [0.99, 1.12]	1.10 [1.01, 1.13]	0.258
FDP (ug/ml)	4.98 [3.20, 7.72]	5.10 [3.50, 7.70]	4.80 [2.90, 7.98]	4.69 [3.09, 6.90]	0.527
TT (s)	16.20 [15.50, 17.00]	16.10 [15.40, 17.10]	16.20 [15.50, 17.00]	16.00 [15.55, 16.75]	0.458
TNF-α (pg/ml)	18.00 [13.85, 24.95]	18.30 [13.30, 25.00]	16.60 [14.30, 22.70]	20.60 [14.70, 27.40]	0.188
**IL-6 (pg/ml)**	**20.80 [7.58**,** 59.60]**	**20.70 [7.73**,** 68.40]**	**17.50 [6.12**,** 43.11]**	**39.20 [13.05**,** 57.10]**	**0.023**
sIL-2R (pg/ml)	2,043.00 [1,346.00, 2,980.00]	2,062.00 [1,353.00, 3,452.00]	2,001.00 [1,326.00, 2,767.00]	2,047.00 [1,490.50, 3,065.00]	0.595
IL-8 (pg/ml)	19.70 [12.75, 37.45]	21.20 [13.70, 41.90]	17.90 [12.00, 31.65]	16.90 [11.70, 30.30]	0.101
IL-10 (pg/ml)	7.84 [2.50, 19.50]	8.58 [2.50, 21.00]	7.23 [2.50, 15.30]	6.90 [2.50, 23.80]	0.425

*Abbreviations*: IVIG, intravenous immunoglobin; KD, Kawasaki disease; cKD, complete Kawasaki disease; iKD, incomplete Kawasaki disease; CALs, coronary artery lesions; Zmax, the maximum Z score; NCAI, number of coronary arteries involved; WBC, white blood cell; N%, neutrophil percent; Hb, haemoglobin; PLT, platelet; CRP, C-reaction protein; ESR, erythrocyte sedimentation rate; Na, serum sodium; ALB, albumin; Tbil, total bilirubin; NT-proBNP, N-Terminal pro-brain natriuretic peptide; PT, prothrombin time; FIB, Fibrinogen; APTT, activated partial thromboplastin time; INR, international Normalized Ratio; FDP, fibrin degradation products; TT, thrombin time; TNF-α, tumor necrosis factor-α; IL-6, interleukin-6; sIL-2R, soluble interleukin-2 receptor; IL-8, interleukin-8; IL-10, interleukin-10

### Extraction and selection of ultrasomics features

Analysis was conducted on 1484 echocardiographic images, from which 5636 ultrasomics features were extracted within the ROIs. These features encompassed 504 first-order features, 56 shape features, 2100 textural features, and 2976 wavelet-based features.

**Fig. 2:**
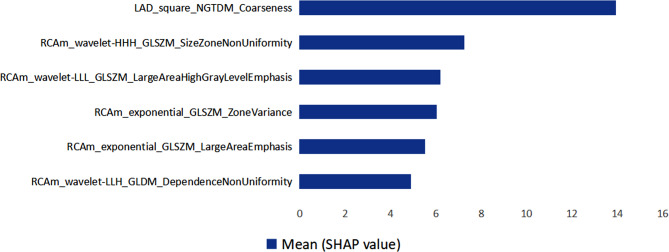
Top 6 ultrasomics features for predicting for CALs progression of KD patients in a descending order of importance. *Abbreviations*: KD, Kawasaki disease; CALs, coronary artery lesions; LAD, left anterior descending artery; RCAm, the middle segment of right coronary artery; NGTDM, neighbouring gray tone difference matrix; GLSZM, gray-level size zone matrix; GLDM, gray level dependence matrix; GLRLM, gray-level run-length matrix.

The performance of ultrasomics features analyzed by nine machine learning algorithms for CALs progression in children with KD are provided in Table 2, including accuracy and AUC. The hyperparameters of the nine machine learning algorithms used in this study are detailed in Table S1. Since the SVM algorithm performed the best on the training set, it was chosen for feature selection. By using the optimal SVM algorithm, the cumulative performance of top 10 factors according to the descending importance was calculated and the top eight important variables had satisfactory prediction power (Table 3). On the final selection of features from the training set, 8 ultrasomics features were final selected. After eliminating the redundancy with correlation coefficients > 0.90, 6 ultrasomics features were finally used for further analysis, including a neighbouring gray tone difference matrix (NGTDM) feature, four gray-level size zone matrix (GLSZM) features, and a gray level dependence matrix (GLDM) feature. To evaluate the contribution of the 6 ultrasomics features selected, the importance of each factor was gauged and ranked, as is illustrated in Fig. 2. Details on the ultrasomics features, which can be available in the description of the Pyradiomics package (https://pyradiomics.readthedocs.io/en/latest/features.html#), are listed in supplementary materials (Table S2).

**Table 2:** Prediction of ultrasomics features analyzed by 9 machine learning algorithms for CALs progression in children with KD

Algorithms	ACC	AUROC
Decision tree	0.80	0.49
SVM	0.83	0.67
RF	0.83	0.52
KNN	0.84	0.48
GBM	0.80	0.54
XGBM	0.78	0.46
LGBM	0.82	0.56
mLP	0.84	0.37
bNB	0.84	0.52

*Abbreviations*: ACC, accuracy; AUROC, area under the receiver operating characteristic curve; SVM, support vector machine; RF, random forest; KNN, K-nearest neighbor; GBM, Gradient boosting machine; XGBM, Extreme gradient boosting machine; LGBM, Light gradient boosting machine; mLP, multi-Layer perceptron; bNB, Bernoulli naive Bayes

**Table 3:** Distributions of AUC, accuracy and precision with the cumulating number of top 10 important factors in an ascending order

Number of top 10 factors in rank	AUROC	ACC	Precision
1	0.60	0.80	0.20
2	0.51	0.73	0.11
3	0.52	0.77	0.10
4	0.52	0.80	0.20
5	0.52	0.80	0.20
6	0.52	0.80	0.20
7	0.53	0.83	0.25
**8**	**0.60**	**0.80**	**0.14**
9	0.60	0.80	0.14
10	0.59	0.78	0.10

*Abbreviations*: AUROC, area under the receiver operating characteristic curve; ACC, accuracy

### Selection of clinical parameters

As shown in the Table 4, the bidirectional stepwise approach selected three variables to construct the clinical model, which were number of coronary arteries involved (OR: 2.40, *P* < 0.01), albumin (ALB) (OR: 0.90, *P* = 0.08), and FIB (OR: 1.30, *P* < 0.01).

**Table 4:** Multivariable logistic regression for clinical variables

Variables	Multivariable logistic regression		
	OR	95% CI	*P* value
**NCAI**	2.44	1.68–3.64	< 0.01
**ALB**	0.90	0.84–0.95	0.08
**FIB**	1.31	1.00-1.73	< 0.01

*Abbreviations*: OR odds ratios; CI confidence interval; NCAI number of coronary arteries involved; ALB albumin; FIB; fibrinogen

### Predictive models construction and evaluation

**Fig. 3:**
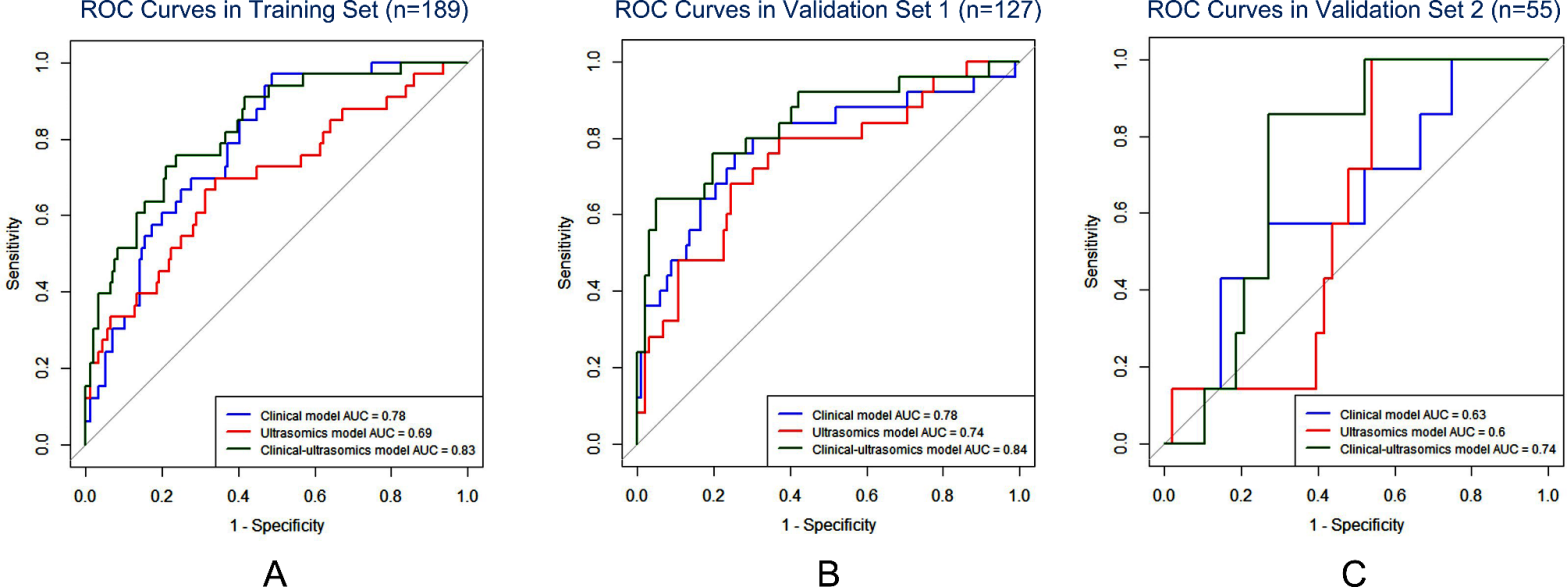
**A**The ROC curves for ultrasomics model, clinical model, and the clinical-ultrasomics model in three sets:**B** ROC curves for three models in validation set 1. ROC curves for three models in validation set 2. *Abbreviations*: ROC, receiver operating characteristics; AUC, area under curve

**Fig. 4:**
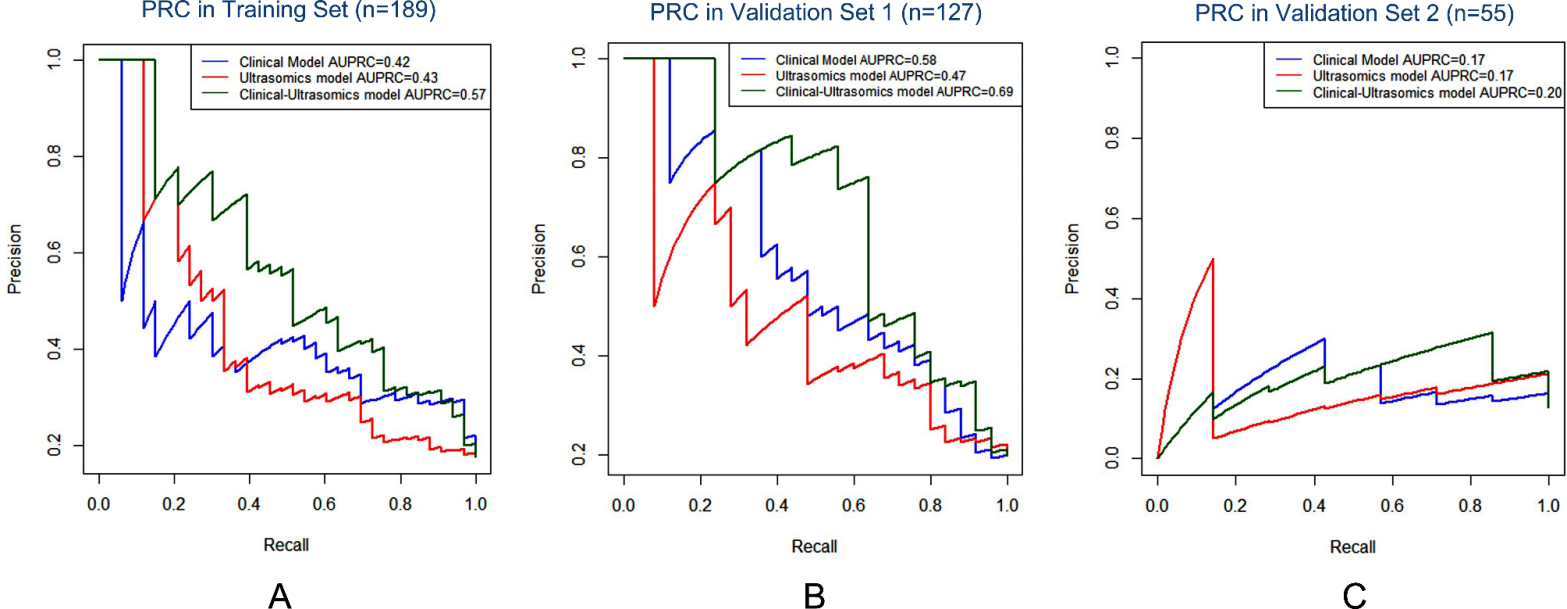
The PRCs for ultrasomics model, clinical model, and the clinical-ultrasomics model in three sets. PRCs for three models in the training set. PRCs for three models in validation set 1. PRCs for three models in validation set 2. *Abbreviations*: PRC, Precision-recall curve; AUPRC, area under the precision-recall curve

**Fig. 5:**
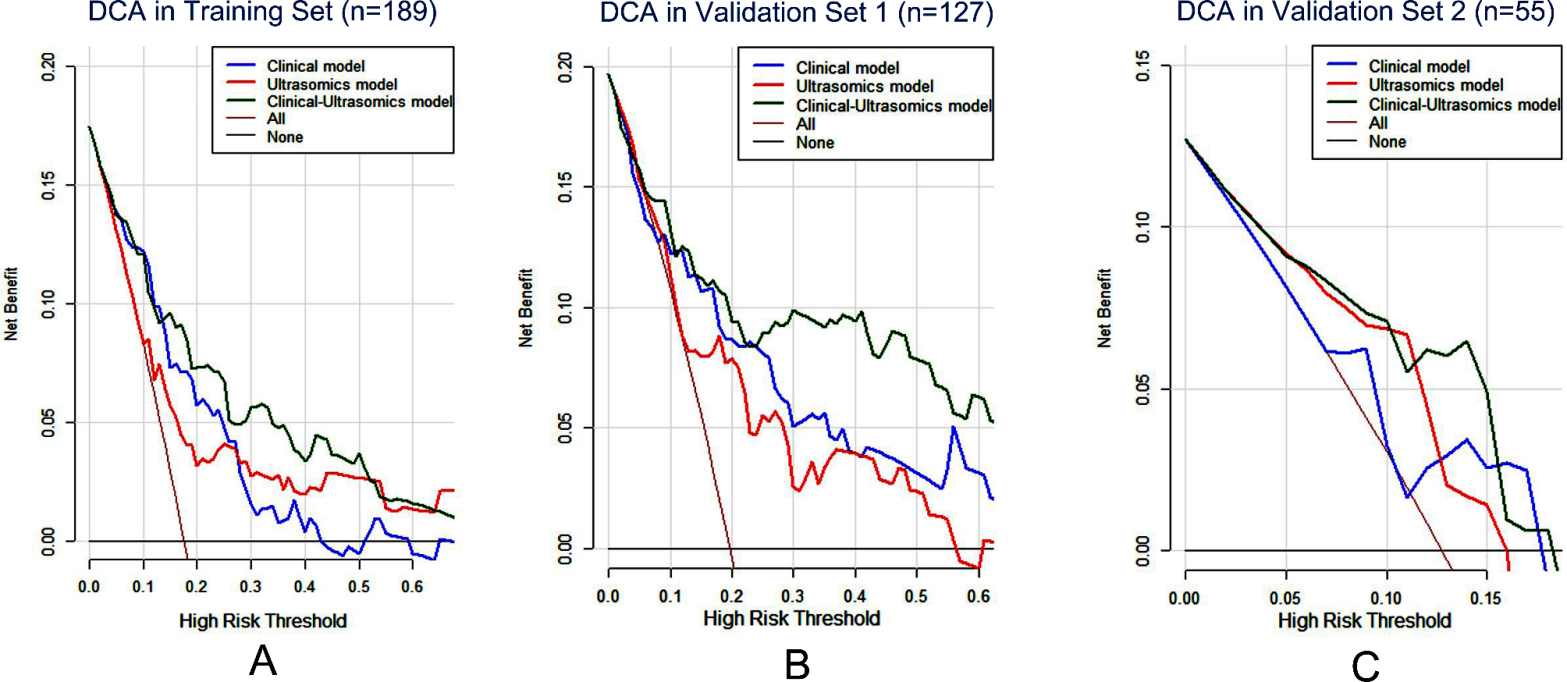
The DCA for ultrasomics model, clinical model, and the clinical-ultrasomics model in three sets. DCA for three models in the training set. DCA for three models in validation set 1. DCA for three models in validation set 2. *Abbreviations*: DCA: Decision curve analysis

The construction and evaluation of clinical model, ultrasomics model and clinical-ultrasomics model in the training set and two validation sets are presented in Figs. 3, 4 and 5. The AUROC of ultrasomics model in the training set (0.69) was found to underperform the clinical model (0.78), while the clinical-ultrasomics model (0.83) exhibited better predictive performance (Fig. 3A). Similar results were observed in the internal validation set and the prospective validation set (Fig. 3B and C), confirming the additional prognostic performance of ultrasomics features. Moreover, the AUPRC and DCA also validated that the clinical-ultrasound omics model outperformed the single clinical model and the ultrasound omics model in both the training set and two validation sets (Fig. 4and Fig. 5).

## Discussion

In the present study, we developed three models to predict the CALs progression from KD onset to the 1-month follow-up, including a model based on clinical parameters, a model based on ultrasomics features, and an integrated model that combined clinical parameters and ultrasomics features. Furthermore, validation across two sets revealed that the clinical-ultrasomics model consistently outperformed both the individual clinical and ultrasomics model.

In our previous study, we developed a predictive model based on clinical parameters to forecast CAL progression at 1 month after KD onset, achieving an AUC value of 0.80^30^. As we know, echocardiography is currently the primary method for assessing CAL in KD, and ultrasomics technology can detect subtle changes in ultrasound images that are not visible to the naked eye. In order to explore whether adding ultrasound imaging information can improve the predictive power of the model, the present study was designed and conducted. The present study showed that the clinical-ultrasomics model achieved an AUC value of 0.84, surpassing the previous model [30]. Additionally, this study broadened the model’s applicability and proved its robustness by validating its performance with a prospective cohort, whereas the applicability of the previous model remained unknown as it was only validated internally. To the best of our knowledge, this is the first study to investigate the value of ultrasomics based on baseline echocardiographic images in predicting the progression of CALs in KD patients.

The clinical model was constructed based on three variables, that is the number of coronary arteries involved, ALB, and FIB. Extensive research has consistently demonstrated an association between a greater number of involved coronary arteries and lower serum ALB levels with CALs progression in KD patients [7, 9, 31, 32], aligning with our own findings. FIB, an acute-phase protein synthesized in the liver under inflammatory or traumatic conditions, is indicative of both hypercoagulation and severe inflammation in patients. Chen et al. [33] observed significantly elevated FIB levels in KD compared to healthy controls. Additionally, Liu et al. [34] demonstrated that plasma FIB concentration in KD patients with CALs was notably higher than in those without CALs. Our study further extended these findings, establishing an association between FIB levels and the progression of CALs in KD.

In this study, SVM was the best machine learning algorithm when predicting the CALs progression of KD patients. SVM constructs a hyperplane concept to classify observations and is the closest machine learning algorithm close to DL, which might account for its optimal performance in this study. The present study identified three texture-based features and three higher-order features obtained by wavelet transformation of the original images. These features partially reflect the texture changes in echocardiograms, suggesting that KD patients at high risk of CAL progression may exhibit texture changes invisible to the naked eye at disease onset. Moreover, the most significant ultrasomics features in the model were all related to the LAD and the proximal or middle segment of RCA. This may be due to the suboptimal visualization of distal coronary segments by echocardiography compared to other segments [1]. Given that our study was designed based on echocardiographic findings, it’s comprehensible that the most significant ultrasomics features were associated with the LAD and RCA. Although most of the ultrasomics features identified in our study were associated with the middle segmentation of RCA, the most significant feature was the LAD related features. Hence, our findings highlight the importance of examining multiple sites of coronary arteries to gather comprehensive information.

The diagnosis of KD primarily relies on clinical findings, laboratory indicators, and echocardiographic observations, all of which lack specificity. While echocardiographic imaging aids in KD diagnosis, it alone is insufficient for KD diagnosis due to the presence of CAD in other febrile diseases [35, 36]. In the present study, the ultrasomics model didn’t surpass the clinical model in predicting CALs progression. This could be attributed to the clinical model encompassed both laboratory indicators (ALB and FIB) and echocardiographic finding (the number of involved coronary arteries), whose information spectrum was broader than the ultrasomics model. Nevertheless, our findings demonstrate that ultrasomics features extracted from echocardiographic images can enhance the prediction of CALs progression when combined with clinical parameters, as evidenced by the superior performance of the clinical-ultrasomics model across three distinct datasets. This trend holds promise as it offers deeper insights into managing KD patients and provides valuable guidance for future research directions. As multiple studies have demonstrated the superiority of CCTA in detecting CALs located in the distal segments and the left circumflex branches of coronary arteries compared with TTE [37, 38], exploring CTCA-based radiomics to identify at-risk KD patients with CALs warrants further investigation.

Several limitations should be acknowledged. Firstly, despite we validated the prediction models with a prospective cohort in our center, the absence of external validation may restrict the generalizability of our findings. Secondly, our reliance on static baseline echocardiographic images provides only a snapshot of the condition at the time of image capture. The utilization of dynamic video records might potentially offer more comprehensive information, thereby enhancing predictive capabilities. Thirdly, as the clinical-ultrasomics model was applied to KD patients with CALs detected by baseline echocardiography to predict the outcome of their CALs at 1 month in the course of KD, the access to high-quality images and the manual segmentation of typical areas of CALs by echocardiography specialist are prerequisites for its application. Fourth, the ROIs of CALs were manually segmented in the present study, which could be subjective and introduce observers’ bias. Semi-automatic or automatic segmentation methods are needed in the future. Last but not least, the imbalance in the number of patients between the two groups within our datasets contributed to the relatively low specificity observed in all three prediction models. Addressing this imbalance by increasing the number of CALs-progressed KD patients to align more closely with the control group’s size may mitigate this issue.

## Conclusions

Our study demonstrated that prediction model involving echocardiogram-based ultrasomics features and clinical parameters has a good predictive efficacy in forecasting CALs progression in 1-month follow-up among KD patients with CALs. This highlights the promising potential of ultrasomics in predicting CALs progression using baseline echocardiographic images, providing clinicians a valuable tool to detect CALs at their early stages and consequently aiding in the tailoring of individualized treatment for KD.

## Electronic supplementary material

Below is the link to the electronic supplementary material.

Supplementary Material 1

## Data Availability

The data that support the findings of this study are available from the corresponding author, upon reasonable request.

## *Abbreviations*

KD: Kawasaki Disease
CALs: Coronary Artery Lesions
LAD: Left Anterior Descending Artery
RCA: Right Coronary Artery
ROC: Receiver Operating Characteristic
PRC: Precision-Recall Curve
DCA: Decision Curve Analysis
AUROC: Area Under The Receiver Operating Characteristic Curve
AUPRC: Area Under The Precision-Recall Curve
