# Supplementary material for: Progression prediction of coronary artery lesions by echocardiography-based ultrasomics analysis in Kawasaki disease
Source: Ital J Pediatr. 2024 Sep 18;50:185. doi: 10.1186/s13052-024-01739-1 (PMC11412030; doi:10.1186/s13052-024-01739-1)
Supplement: Supplementary file 1 — Supplementary Material 1 [file 13052_2024_1739_MOESM1_ESM.docx]

**Table S1** Hyper-parameters of 9 machine learning algorithms adopted in this study

| **Algorithms** | **Hyperparameters** |
| --- | --- |
| Decision tree | methods: anova, poisson, class or exp.  cp values: 0.01 to 0.1 by 0.01.  split methods: information or gini. |
| SVM | classification type: C-classification, nu-classification, one-classification, eps-regression or nu-regression.  tolerance: 0.001 to 0.1 by 0.001.  epsilon in the insensitive-loss function: 0.01 to 0.1 by 0.01.  gamma: 0.01 to 0.1 by 0.01. |
| RF | number of trees: 100 to 10000 by 100. |
| KNN | number of neighbors considered: 3 to 9 by 2.  minkowski distance: 0.1 to 10 by 0.1.  kernel: rectangular, triangular, epanechnikov, biweight, triweight, cos, inv, Gaussian, rank or optimal. |
| GBM, XGBM, LGBM | total number of trees: 10 to 10000 by 10.  maximum depth of each tree: 1 to 10 by 1.  shrinkage parameter: 0.01 to 1.0 by 0.01.  cross-validation folds: 2 to 20 by 1. |
| MLP | hidden layers: 1 to 20 by 1.  input items: 2 to 30. |
| bNB | positive double controlling Laplace smoothing: 0 to 10 by 1.  value replacing cells with probabilities within eps range: 0.001 to 0.1 by 0.001. |

**Abbreviations: SVM, support vector machine; RF, random forest; KNN, K-nearest neighbor; GBM, Gradient boosting machine; XGBM, Extreme gradient boosting machine; LGBM, Light gradient boosting machine; MLP, multi-layer perceptron; bNB, Bernoulli naive Bayes.**

**Table S2** Details regarding these ultrasomics features

| **Type** | **Features** | **Description** |
| --- | --- | --- |
| NGTDM | Coarseness, Contrast, Busyness, Complexity, Strength | The quantification of the difference between a gray value and the average gray value of its neighbors within distance. |
| GLSZM | Small Area Emphasis, Large Area Emphasis, Gray Level Non-Uniformity, Gray Level Non-Uniformity Normalized, Size-Zone Non-Uniformity, Size-Zone Non-Uniformity Normalized, Zone Percentage, Gray Level Variance, Zone Variance, Zone Entropy, Low Gray Level Zone Emphasis, High Gray Level Zone Emphasis, Small Area Low Gray Level Emphasis, Small Area High Gray Level Emphasis, Large Area Low Gray Level Emphasis, Large Area High Gray Level Emphasis | The quantification of the gray level zones in images. The gray level zone is defined as the number of connected voxels that share the same gray level intensity. |
| GLDM | Small Dependence Emphasis, Large Dependence Emphasis, Gray Level Non-Uniformity, Dependence Non-Uniformity, Dependence Non-Uniformity Normalized, Gray Level Variance, Dependence Variance, Dependence Entropy, Low Gray Level Emphasis, High Gray Level Emphasis, Small Dependence Low Gray Level Emphasis, Small Dependence High Gray Level Emphasis, Large Dependence Low Gray Level Emphasis,Large Dependence High Gray Level Emphasis. | The quantification of gray level dependencies in images. A gray level dependency is defined as a the number of connected voxels within distance that are dependent on the center voxel. |

**Abbreviations: GLDM, gray-level dependence matrix; GLSZM, gray-level size zone matrix; NGTDM, neighboring gray-tone difference matrix**
